# An SNP Marker Predicts Colorectal Cancer Outcomes with 5-Fluorouracil-Based Adjuvant Chemotherapy Post-Resection

**DOI:** 10.3390/ijms25126642

**Published:** 2024-06-17

**Authors:** Hao Chien, Yu-De Chu, Yi-Ping Hsu, Chau-Ting Yeh, Ming-Wei Lai, Ming-Ling Chang, Siew-Na Lim, Chun-Wei Chen, Wey-Ran Lin

**Affiliations:** 1 Department of Hepatology and Gastroenterology, Linkou Chang Gung Memorial Hospital, Taoyuan 333423, Taiwan; chien.aaron@gmail.com (H.C.); chautingy@gmail.com (C.-T.Y.); mlchang8210@gmail.com (M.-L.C.); 2 Liver Research Center, Linkou Chang Gung Memorial Hospital, Taoyuan 333423, Taiwan; yudechu19871003@gmail.com (Y.-D.C.); 014993irb@gmail.com (Y.-P.H.); mingweilai@gmail.com (M.-W.L.); 3 Institute of Stem Cell and Translational Cancer Research, Linkou Chang Gung Memorial Hospital, Taoyuan 333323, Taiwan; 4 Division of Pediatric Gastroenterology, Department of Pediatrics, Linkou Chang Gung Memorial Hospital, Taoyuan 333423, Taiwan; 5 Department of Neurology, Linkou Chang Gung Memorial Hospital, Taoyuan 333423, Taiwan; siewna@cgmh.org.tw

**Keywords:** genome-wide association study, colorectal cancer, adjuvant chemotherapy, therapeutic predictor, 5-fluorouracil

## Abstract

Colorectal cancer (CRC) is a global health concern, necessitating adjuvant chemotherapy post-curative surgery to mitigate recurrence and enhance survival, particularly in intermediate-stage patients. However, existing therapeutic disparities highlight the need for biomarker-guided adjuvant chemotherapy to achieve better CRC inhibition. This study explores the molecular mechanisms underlying the inhibition of CRC through a genome-wide association study (GWAS) focused on 5-fluorouracil (5-FU)-based adjuvant therapy in intermediate-stage CRC patients, a domain previously unexplored. We retrospectively included 226 intermediate-stage CRC patients undergoing surgical resection followed by 5-FU-based adjuvant chemotherapy. The exploration cohort comprised 31 patients, and the validation cohort included 195 individuals. Genotyping was carried out using either Axiom Genome-Wide TWB 2.0 Array Plate-based or polymerase chain reaction-based methods on genomic DNA derived from collected tissue samples. Statistical analyses involved descriptive statistics, Kaplan–Meier analyses, and Cox proportional hazard analyses. From the GWAS, potential genetic predictors, *GALNT14*-rs62139523 and *DNMBP*-rs10786578 genotypes, of 5-FU-based adjuvant therapy following surgery in intermediate-stage CRC patients were identified. Validation in a larger cohort of 195 patients emphasized the predictive significance of *GALNT14*-rs62139523 genotypes, especially the “A/G” genotype, for improved overall and progression-free survival. This predictive association remained robust across various subgroups, with exceptions for specific demographic and clinical parameters such as age < 58 years old, CEA ≤ 2.5 ng/mL, tumor diameter > 44.0 mm, and tumor-free margin ≥ 50 mm. This study identifies that the *GALNT14*-rs62139523 “A/G” genotype modulates therapeutic outcomes, establishing it as a promising biomarker for predicting favorable responses to 5-FU-based adjuvant chemotherapy in intermediate-stage CRC patients, although further investigations are needed to detail these mechanisms.

## 1. Introduction

Colorectal cancer (CRC) remains a significant global health concern, contributing to high cancer-related mortality rates, even in developed countries like Taiwan. In 2020, there were nearly one million new CRC cases, resulting in approximately 5.7 million deaths worldwide [1]. Despite advancements in diagnostics, a considerable number of CRC cases are diagnosed at intermediate to advanced stages, limiting the effectiveness of surgical resection and increasing the risk of post-surgical recurrence and metastasis. This highlights the necessity for additional therapeutic strategies [2].

At diagnosis, around 80% of CRC patients have localized and resectable disease, leading to varying five-year survival rates of approximately 90% for stage I, 70–80% for stage II, and 40–65% for stage III. Notably, the risk of recurrence is stage-dependent, with estimated rates of 30% for stage II and 50% for stage III, mainly within the first two years post-surgery [3].

To mitigate the risk of recurrence and enhance survival rates, adjuvant chemotherapy is commonly recommended following curative surgery to inhibit CRC progression, particularly for intermediate-stage patients, including stage III and high-risk stage II individuals with specific negative prognostic indicators [4]. The combination of fluoropyrimidine or its derivatives, such as 5-fluorouracil (5-FU) and Xeloda, with oxaliplatin has become a fundamental aspect of international adjuvant treatment guidelines [5]. While debates exist regarding the efficacy of adjuvant therapy due to observed disparities in therapeutic outcomes among CRC patients, it undeniably benefits a substantial proportion of patients [6,7].

Variations in the outcomes of adjuvant chemotherapy are often attributed to factors like tumor clonal heterogeneity and genetic differences, complicating treatment decision making [8]. This underscores the urgent need for the development of biomarker-guided adjuvant chemotherapy for CRC, specifically focusing on genetic biomarkers.

Numerous studies have illuminated various genetic variations associated with the therapeutic effectiveness in CRC, offering insights into potential genetic biomarkers relevant to CRC treatment. These include therapies targeting epithelial growth factor receptor (EGFR) [9,10], immunotherapies [11], and chemotherapy regimens involving agents such as 5-FU [12,13,14,15] and oxaliplatin [16,17,18,19]. Notably, a comprehensive genome-wide association study (GWAS) has revealed single nucleotide polymorphism (SNP) biomarkers that may predict the clinical outcomes of both intermediate and advanced CRC patients undergoing oxaliplatin-based adjuvant chemotherapy [19]. However, a comprehensive study, especially in the context of a GWAS, investigating 5-FU-based adjuvant therapy, is currently lacking.

An analogue of uracil with a fluorine atom at the C-5 position replacing hydrogen, 5-FU is a widely used antimetabolite drug for cancer treatment, particularly for inhibiting CRC. The molecular mechanisms of CRC inhibition via 5-FU have been well documented over the past decades [20,21]. Specifically, 5-FU rapidly enters cells using the same facilitated transport mechanism as uracil [22]. Once inside, 5-FU is converted intracellularly into several key active metabolites: fluorodeoxyuridine monophosphate (FdUMP), fluorodeoxyuridine triphosphate (FdUTP), and fluorouridine triphosphate (FUTP). These active metabolites disrupt DNA and RNA synthesis and inhibit the action of thymidylate synthetase (TS), exerting anticancer effects through TS inhibition and incorporation of metabolites into RNA and DNA [20].

In the 5-FU catabolism and clearance process, the rate-limiting enzyme is dihydropyrimidine dehydrogenase (DPYD), which converts more than 80% of 5-FU to dihydrofluorouracil (DHFU) in the liver [20]. Therefore, reducing DPYD activity in the liver can enhance 5-FU-mediated cytotoxicity through decreasing 5-FU clearance [23]. On the other hand, the anabolism of 5-FU, which is required for the synthesis of key active metabolites related to 5-FU-mediated cytotoxicity, occurs under normal physiological conditions in cells other than hepatocytes. TS catalyzes the reductive methylation of deoxyuridine monophosphate (dUMP) to deoxythymidine monophosphate (dTMP), using the reduced folate 5,10-methylenetetrahydrofolate (CH_2_FH_4_) as the methyl donor. This reaction provides the sole de novo source of thymidylate, which is necessary for DNA replication and repair. Methylenetetrahydrofolate reductase (MTHFR), a key regulatory enzyme, catalyzes a unidirectional reaction involved in intracellular folate metabolism, irreversibly converting CH_2_FH_4_, required for purine and thymidine synthesis, into 5-methyltetrahydrofolate (CH_3_FH_4_). This directs the folate pool towards the remethylation of homocysteine to methionine, representing the main determinant of intracellular CH_2_FH_4_ concentration [21]. The 5-FU metabolite FdUMP binds to the nucleotide-binding site of TS, forming a stable ternary complex with the enzyme and CH_2_FH_4_, thereby blocking the binding of the normal substrate dUMP and inhibiting dTMP synthesis, effectively blocking DNA replication [21]. Additionally, the 5-FU metabolite FUTP can be extensively incorporated into RNA, disrupting normal RNA processing and function, affecting gene expression and impacting the maintenance of basic conditions for cell survival [24].

Various mutations and SNPs have been identified that correlate with the efficacy of 5-FU in CRC patients, primarily residing in genes crucial to 5-FU metabolism, potentially explaining their impact on therapeutic outcomes. MTHFR, a significant enzyme in 5-FU metabolism, exhibits an improved response in the presence of C677T mutations, while A1298C genotypes appear to have a negligible effect [12]. Additionally, polymorphisms in the TS gene, a direct target of 5-FU, were associated with tumor T stage and found to be indicative of patient survival in stage III CRC patients undergoing 5-FU-based adjuvant therapy [13]. However, these TS gene variants do not consistently predict disease-free survival (DFS) in stage III CRC patients receiving 5-FU adjuvant chemotherapy [14]. Another exploration into polymorphisms of the orotate phosphoribosyl transferase (OPRT) gene, a key enzyme in 5-FU phosphoribosylation crucial for inhibiting tumor growth, did not reveal significant correlations with 5-FU sensitivity [15].

While these preceding studies hint at the potential of genetic variations within specific genes to predict the therapeutic efficacy of 5-FU-based adjuvant chemotherapy in CRC patients, the lack of a comprehensive GWAS exploring the genetic predictors of 5-FU-based adjuvant therapy in intermediate-stage CRC patients necessitates further investigation. Therefore, this study seeks to identify genetic biomarkers, particularly SNP-based markers, predicting the outcomes of 5-FU-based adjuvant chemotherapy, through conducting a GWAS using the Axiom Genome-Wide TWB 2.0 Array Plate [25].

## 2. Results

### 2.1. Study Design

**Figure 1:**
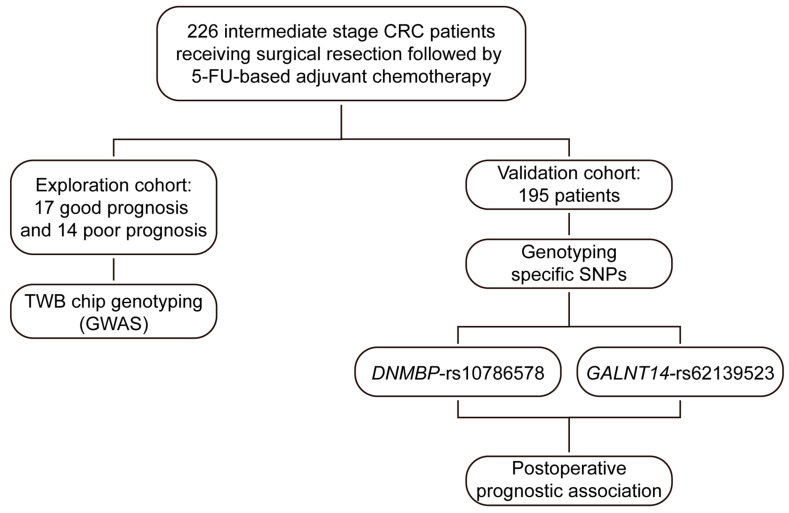
Overview of the study design. This study comprised two cohorts: the exploration cohort and the validation cohort. The exploration cohort involved 17 patients with a favorable prognosis and 14 with a poor prognosis, subjected to a genome-wide association study using the Axiom Genome-Wide TWB 2.0 Array Plate (TWB chip). The validation cohort consisted of 195 patients, selected for genotyping of *GALNT14*-rs62139523 and *DNMBP*-rs10786578 based on the results from the exploration cohort genome-wide association study. Subsequently, the study evaluated the associations between the genotypes of these two candidates and clinical outcomes in the validation cohort.

This study aimed to conduct a comprehensive GWAS analysis to explore the genetic predictors of 5-FU-based adjuvant therapy in intermediate-stage CRC patients, utilizing the Axiom Genome-Wide TWB 2.0 Array Plate. As illustrated in Figure 1, this study retrospectively included two independent cohorts: the exploration and validation cohorts. The exploration cohort comprised a total of 31 patients, with 17 exhibiting a favorable prognosis and 14 demonstrating a poor response to curative surgery followed by 5-FU-based adjuvant chemotherapy.

Previous research summarizing data from 18 randomized control trials involving 20,898 CRC patients demonstrated that adjuvant chemotherapy significantly improved disease-free survival (DFS) in patients with resected stage III and selected stage II CRC (combined as intermediate stages). This benefit was primarily seen in the first two years of treatment and translated into long-term overall survival (OS) benefits. Notably, recurrence rates were low after five years and minimal after eight years, indicating that long-term follow-up for recurrence is of little value beyond this period [3]. In the current study, patients with a good prognosis were defined as those with no recurrence/metastasis or CRC-related disease occurring more than five years after treatment. Conversely, patients with a poor response were those who experienced recurrence or metastasis within two years after treatment, a period during which adjuvant chemotherapy should provide the most significant benefit [3].

Genomic DNA derived from subjects in this cohort underwent GWAS analysis using the Axiom Genome-Wide TWB 2.0 Array Plate to identify genetic variants and potential candidates for predicting the efficacy of 5-FU-based adjuvant chemotherapy after curative surgery in CRC patients, distinguishing between those with favorable and poor treatment responses.

The second cohort, consisting of 195 intermediate-stage CRC patients, was included to validate the potential of the SNP candidates identified from the exploration cohort in predicting the therapeutic efficacy of 5-FU-based adjuvant chemotherapy following curative resection. After genotyping the specific SNP candidates, correlations between genotypes and OS and progression-free survival (PFS) were analyzed to assess the effectiveness of these candidates in relation to the clinical outcomes in patients receiving 5-FU-based adjuvant chemotherapy post-curative resection.

### 2.2. Comparison of Baseline Characteristics in the Exploration Cohort of CRC Patients

The GWAS analysis utilized genomic DNA obtained from the 31 subjects within the exploratory cohort. Among them, 17 exhibited a favorable therapeutic response, while the remaining 14 were classified as having a poor therapeutic response to 5-FU-based adjuvant chemotherapy following curative surgical resection. Their baseline clinicopathological characteristics are summarized in Table 1. Between these two groups, it was observed that only circulating CEA levels and no other parameters showed a significant difference, with higher levels in those categorized under poor prognosis (*p* = 0.002). This finding underscores the prognostic significance of pre-operative circulating carcinoembryonic antigen (CEA) levels in association with prognosis in CRC patients undergoing chemotherapy, as reported previously [26,27].

### 2.3. GWAS Analysis Identifies GALNT14-rs62139523 and DNMBP-rs10786578 Genotypes as Potential Genetic Predictors

**Figure 2:**
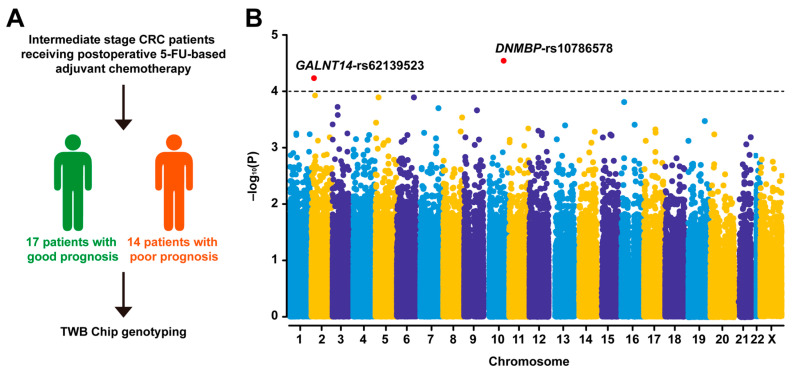
Identification of potential predictive biomarkers, *GALNT14*-rs62139523 and *DNMBP*-rs10786578, for clinical outcomes in CRC patients undergoing curative surgical resection followed by 5-FU-based adjuvant chemotherapy: (**A**) Depiction of the subjects included in the Axiom Genome-Wide TWB 2.0 Array Plate (TWB chip)-based genome-wide association study; (**B**) Manhattan plot illustrating genomic coordinates on the x-axis and the negative logarithm of the association *p*-value (−log_10_(*p*-value)) for each SNP on the y-axis, with each dot representing an SNP. The red dots highlight the most significant differences in the genotypes of *GALNT14*-rs62139523 and *DNMBP*-rs10786578 observed between patients with favorable and unfavorable responses.

After genotyping using the TWB chip, the resulting genotyping data between good and poor response groups were compared using the Chi-square test (Figure 2A). Upon comparative analysis of genotype differences between these groups, a list of SNPs, including *GALNT14*-rs62139523, *PLEKHH2*-rs13408620, *PLEKHH2*-rs6544681, *PLEKHH2*-rs6544679, *FHIT*-rs689497, rs13078044, *PTPRG*-rs657813, rs28794939, rs13171304, *LOC124901426*-rs62425913, rs10259447, rs984449, *DMNBP*-rs10786578, *NTM*-rs10791160, rs7133799, rs7318264, *LITAF*-rs2868424, rs2542671, *MAPT*-rs2435213, and rs1871045, were identified as significantly different between the two groups, with *p*-values smaller than 0.001 (Table 2). The functional consequences of these variants were classified as non-coding transcript variant, intron variant, intergenic, and synonymous variant, with none resulting in a change in the open reading frames of their located genes. Among these candidates, the most significant differences between the two groups were observed in the genotypes of *GALNT14*-rs62139523 and *DMNBP*-rs10786578 (*p* = 0.0000585 and 0.0000285, respectively) (Table 2 and Figure 2B). Notably, the *GALNT14*-rs62139523 genotype was predominantly heterozygous “A/G” in patients with a favorable response (“A/G” = 14 vs. “A/A” + “G/G” = 3), contrasting with the homozygous “A/A” or “G/G” genotype in those with an unfavorable response (“A/G” = 1 vs. “A/A” + “G/G” = 13). Similarly, the *DNMBP*-rs10786578 genotype was primarily heterozygous “C/T” in patients with a favorable response (“C/T” = 15 vs. “A/A” + “G/G” = 2), while it was either homozygous “C/C” or “T/T” in those with an unfavorable response (“A/G” = 1 vs. “A/A” + “G/G” = 13).

### 2.4. Comparison of Baseline Characteristics in the Validation Cohort of CRC Patients

To validate the efficacy of *GALNT14*-rs62139523 and *DNMBP*-rs10786578 genotypes as potential biomarkers for predicting the therapeutic response to 5-FU-based adjuvant chemotherapy following curative surgical resection in intermediate-stage CRC patients, an additional cohort comprising 195 patients was retrospectively enrolled as the validation cohort. After genotyping both *GALNT14*-rs62139523 and *DNMBP*-rs10786578, patients were subgrouped into two groups based on *DNMBP*-rs10786578 “C/T” and “non-C/T” genotypes or *GALNT14*-rs62139523 “A/G” and “non-A/G” genotypes to compare potential differences in baseline characteristics among patients. As presented in Table 3, no significant differences in baseline parameters were observed between patients with *DNMBP*-rs10786578 “C/T” and “non-C/T” genotypes. On the other hand, a higher proportion of males with the *GALNT14*-rs62139523 “non-A/G” genotype was found compared with those with “A/G” genotype (*p* = 0.002). Moreover, a greater incidence of poorly differentiated histology was also observed in association with patients with the “non-A/G” genotype (*p* = 0.005).

### 2.5. Association of GALNT14-rs62139523, but Not DNMBP-rs10786578, Genotypes with Prognosis in Intermediate-Stage CRC Patients Undergoing Surgical Resection Followed by 5-FU-Based Adjuvant Chemotherapy

To explore the association between *DNMBP*-rs10786578 and *GALNT14*-rs62139523 genotypes, alongside other clinical parameters, and OS, Cox proportional hazards analysis was conducted. As presented in Table 4, in the univariate analysis, patients with *GALNT14*-rs62139523 “A/G” genotype (*p* = 0.001), lower T stage (pT1 to pT3) (*p* = 0.010), lymph node metastasis (*p* = 0.011), and lower circulating CEA levels (*p* < 0.001) were associated with favorable OS. In the subsequent multivariate analysis, all four factors emerged as independent predictors of OS: *GALNT14*-rs62139523 “A/G” genotype (*p* = 0.008), lower T stage (pT1 to pT3) (*p* = 0.022), lymph node metastasis (*p* = 0.002), and lower circulating CEA levels (*p* < 0.001). Notably, *DNMBP*-rs10786578 genotypes were not associated with OS (*p* = 0.114).

Similarly, upon examining the association between *DNMBP*-rs10786578 and *GALNT14*-rs62139523 genotypes, alongside other clinical parameters, and PFS, the univariate analysis revealed that patients with *GALNT14*-rs62139523 “A/G” genotype (*p* = 0.003), larger tumor diameter (*p* = 0.040), lower T stage (pT1 to pT3) (*p* = 0.038), and lower circulating CEA levels (*p* < 0.001) were associated with better PFS. In the multivariate analysis, all four factors were independent predictors of PFS: *GALNT14*-rs62139523 “A/G” genotype (*p* = 0.006), larger tumor diameter (*p* = 0.003), lower T stage (pT1 to pT3) (*p* = 0.004), and lower circulating CEA levels (*p* < 0.001). However, *DNMBP*-rs10786578 genotypes were not associated with PFS (*p* = 0.211).

**Figure 3:**
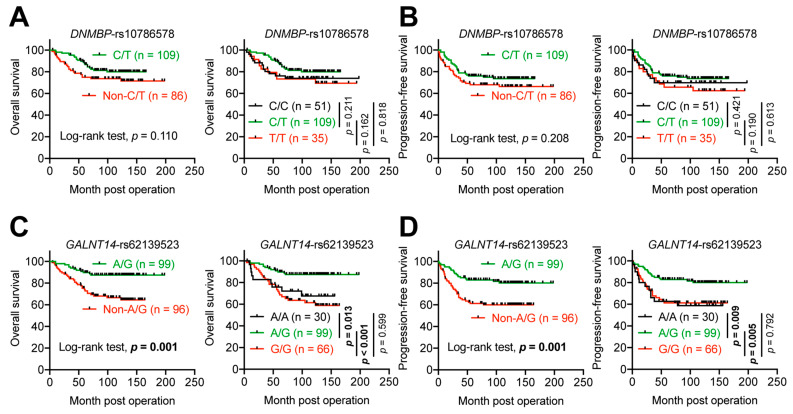
Associations of *GALNT14*-rs62139523 and *DNMBP*-rs10786578 genotypes with clinical outcomes in CRC patients undergoing curative surgical resection followed by 5-FU-based adjuvant chemotherapy. Kaplan–Meier analysis illustrating the associations of *DNMBP*-rs10786578 genotypes with (**A**) overall survival and (**B**) progression-free survival, with patients stratified into either two groups (“C/T” vs. “non-C/T”) or three groups (“C/C” vs. “C/T” vs. “T/T”). Kaplan–Meier analysis demonstrating the associations of *GALNT14*-rs62139523 genotypes with (**C**) overall survival and (**D**) progression-free survival, with patients stratified into either two groups (“A/G” vs. “non-A/G”) or three groups (“A/A” vs. “A/G” vs. “G/G”). All *p*-values were determined using the log-rank test. Bold *p*-values indicate statistical significance.

To confirm the associations of the DNMBP-rs10786578 or GALNT14-rs62139523 genotypes with OS and PFS, Kaplan–Meier analysis was applied. In line with the findings from the Cox proportional hazard analysis (Table 4), *DNMBP*-rs10786578 genotypes exhibited no significant associations with either OS or PFS, regardless of patient stratification into two groups (“C/T” and “non-C/T”) or three groups (“C/C”, “C/T”, and “T/T”) (Figure 3A,B). Although a trend towards improved OS and PFS was observed in patients with the “C/T” genotype, the difference did not reach statistical significance.

Conversely, when patients were stratified based on *GALNT14*-rs62139523 genotypes, those withthe “A/G” genotype demonstrated significantly better OS (Figure 3C, *p* = 0.001, compared with “non-A/G”; *p* = 0.013, compared with “A/A”; *p* < 0.001, compared with “G/G”) and PFS (Figure 3D, *p* = 0.001, compared with “non-A/G”; *p* = 0.009, compared with “A/A”; *p* = 0.005, compared with “G/G”). This re-emphasizes the predictive value of *GALNT14*-rs62139523 “A/G” genotype for favorable OS and PFS in intermediate-stage CRC patients receiving surgical resection followed by 5-FU-based adjuvant chemotherapy.

### 2.6. Exploring the Predictive Role of GALNT14-rs62139523 “A/G” Genotype in Favorable OS and PFS across Subgroups in Intermediate-Stage CRC Patients Undergoing Surgical Resection Followed by 5-FU-Based Adjuvant Chemotherapy

**Figure 4:**
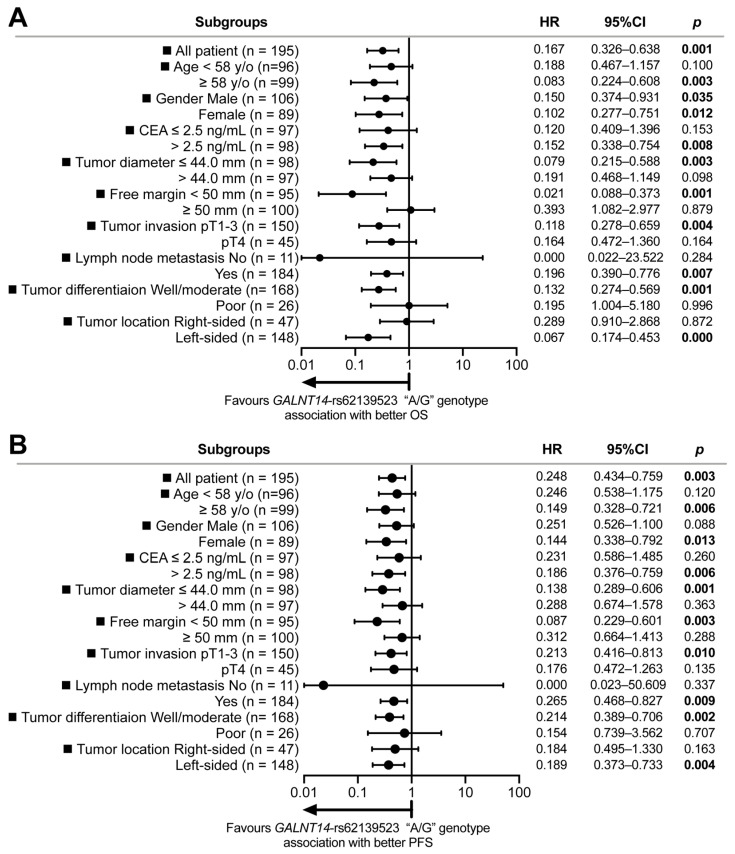
Subgroup analysis reveals optimal subgroups for utilizing *GALNT14*-rs62139523 “A/G” genotype as a predictor for CRC patients undergoing curative surgical resection followed by 5-FU-based adjuvant chemotherapy: (**A**) identification of subgroups where *GALNT14*-rs62139523 “A/G” genotype was associated with improved overall survival; (**B**) identification of subgroups where *GALNT14*-rs62139523 “A/G” genotype was associated with enhanced progression-free survival.

To further investigate the predictive utility of *GALNT14*-rs62139523 “A/G” genotype for favorable OS and PFS in intermediate-stage CRC patients undergoing surgical resection followed by 5-FU-based adjuvant chemotherapy, subgroup analyses were conducted using Cox proportional hazards models across various subgroups. As illustrated in the forest plot in Figure 4, the hazard ratios consistently favored the left side of the perpendicular reference line in most subgroups, indicating an association between the *GALNT14*-rs62139523 “A/G” genotype and improved OS and PFS.

However, specific subgroups, including patients with age < 58 years old, CEA ≤ 2.5 ng/mL, tumor diameter > 44.0 mm, tumor free margin ≥ 50 mm, tumor invasion pT4, no lymph node metastasis, poorly differentiated tumor, and right-sided tumor, showed no significant association of the *GALNT14*-rs62139523 “A/G” genotype with OS. Additionally, in certain subgroups, including patients with age < 58 years old, male gender, CEA ≤ 2.5 ng/mL, tumor diameter > 44.0 mm, tumor free margin ≥ 50 mm, tumor invasion pT4, no lymph node metastasis, poorly differentiated tumors, and right-sided tumors, no significant association with PFS was observed.

In some of these subgroups, such as tumor invasion pT4 (*n* = 45), no lymph node metastasis (*n* = 11), poorly differentiated tumor (*n* = 26), and right-sided tumor (*n* = 47), the small case numbers may have contributed to the lack of statistical significance. Nevertheless, in the majority of subgroups, parameters like age, CEA levels, tumor diameter, and tumor free margin seemed to influence the predictive value of *GALNT14*-rs62139523 “A/G” genotype for better OS and PFS in intermediate-stage CRC patients receiving surgical resection followed by 5-FU-based adjuvant chemotherapy. This suggests that the predictive potential of the *GALNT14*-rs62139523 “A/G” genotype holds true in most subgroups, except for those with age < 58 years old, CEA ≤ 2.5 ng/mL, tumor diameter > 44.0 mm, and tumor free margin ≥ 50 mm.

In conclusion, this study conducted a comprehensive GWAS analysis to explore genetic predictors of 5-FU-based adjuvant therapy in intermediate-stage CRC patients, identifying *GALNT14*-rs62139523 and *DNMBP*-rs10786578 genotypes as potential predictors. The subsequent validation in a larger cohort of 195 patients underscored the predictive significance of *GALNT14*-rs62139523 genotypes, particularly the “A/G” genotype, in terms of OS and PFS. Notably, this predictive association was robust across various subgroups, except for specific demographic and clinical parameters such as age < 58 years old, CEA ≤ 2.5 ng/mL, tumor diameter > 44.0 mm, and tumor free margin ≥ 50 mm. These findings emphasize the promising role of *GALNT14*-rs62139523 “A/G” genotype as a biomarker for predicting favorable outcomes in intermediate-stage CRC patients undergoing surgical resection followed by 5-FU-based adjuvant chemotherapy, with attention to specific subgroup characteristics.

## 3. Discussion

Although adjuvant chemotherapeutic treatment is currently highly recommended for patients with high-risk stage II and stage III CRC, ongoing debates surround their therapeutic effects [28]. Nevertheless, it is well documented that 5-FU-based adjuvant chemotherapy confers a survival advantage to a sustained proportion of stage II and III CRC patients [6,7,29]. Notably, only up to 30% of patients truly benefit from this adjuvant treatment, with 50% achieving cure through surgery alone and 20% experiencing disease recurrence despite adjuvant therapy [30]. Given these disparities in clinical outcome in patients undergoing 5-FU-based adjuvant chemotherapy after surgical resection, there is an urgent need to identify biomarkers for personalized adjuvant therapy [31]. Despite increasing evidence suggesting potential biomarkers for guiding such treatment [31], our ability to predict prognosis and treatment efficacy remains suboptimal. Accordingly, this study is dedicated to identifying potential SNP biomarkers capable of predicting prognosis in this patient group.

To identify potential SNP biomarkers, GWAS analysis is a frequently used technique for discovering genetic factors that influence diseases and therapeutic efficacy [32]. During this analysis, hundreds of thousands or even millions of genetic loci are probed in a set of cases and controls from included cohorts. The association between genotype and phenotype for each genetic locus is typically measured using a Chi-squared (or Fisher’s exact) test. Due to the multiple comparisons inherent in GWAS, a more stringent significance threshold, such as Bonferroni correction, may be employed, resulting in “genome-wide” significant variants with a *p*-value smaller than 5 × 10^−8^, particularly for common variants [33]. Although using this stringent *p*-value threshold has been remarkably successful in limiting false-positive findings and has led to robust and reproducible results, there has been ongoing discussion about revisiting and relaxing this threshold [34]. The stringent *p*-value threshold may mask the identification of many true genetic signals, especially for those with minor allele frequency, despite its benefit of rigorous false-positive control [35]. Additionally, the *p*-value can be affected by the sample size and the total number of SNPs detected [35,36]. In this study, because stage II/III CRC patients are not always required to receive adjuvant chemotherapy, particularly 5-FU-based therapy, the sample size in the exploration cohort was relatively small, which in turn affected the GWAS *p*-value threshold.

Following GWAS analysis and meticulous validation, this study has uncovered a significant association between the *GALNT14*-rs62139523 “A/G” genotype and improved OS and PFS across all enrolled patients, while the *DNMBP*-rs10786578 “C/T” genotype, another candidate SNP biomarker, did not exhibit a significant correlation with survival outcomes in the validation cohort (Table 4). Remarkably, the association between the *GALNT14*-rs62139523 “A/G” genotype and favorable clinical outcomes persisted even after adjusting for other clinicopathological factors in multivariate analysis. Consistent with previous studies [37,38,39], other clinical parameters, such as deep tumor invasion (pT4), lymph node metastasis (at least N1), and elevated CEA levels, were significantly linked to poor OS, while tumor size (diameter), deep tumor invasion (pT4), and CEA levels were correlated with poor PFS. Therefore, the *GALNT14*-rs62139523 “A/G” genotype emerges as a potential biomarker for predicting outcomes in stage II and III colon cancer patients undergoing curative surgery followed by 5-FU-based adjuvant chemotherapy.

To our knowledge, there has been no previous report elucidating the role of *GALNT14*-rs62139523 genotypes in association with clinical events. We are the first to underscore its prognostic significance in stage II and III CRC patients undergoing curative surgery followed by 5-FU-based adjuvant chemotherapy. Notably, although no reports have specifically addressed this particular SNP, other genetic variations within the *GALNT14* gene have demonstrated associations with diverse therapeutic responses in different cancers. These encompass responses to transcatheter arterial chemoembolization in intermediate-stage hepatocellular carcinoma patients [40], responses to systemic chemotherapy and targeted therapy in advanced hepatocellular carcinoma patients [41,42,43,44], responses to concurrent chemoradiotherapy in advanced esophageal squamous cell carcinoma [45], postoperative prognosis in patients with pancreatic ductal adenocarcinoma [46], cholangiocarcinoma [47], advanced gastric cancer [48], head and neck cancer [49], and hepatocellular carcinoma [50], as well as responses to oxaliplatin-based adjuvant chemotherapy in stage III CRC patients [51]. Moreover, mutations identified in the *GALNT14* gene have demonstrated it to be a potential neuroblastoma predisposition gene [52]. These cumulative findings strongly suggest that genetic variations within or near the *GALNT14* gene may wield substantial effects on cancer behavior, thereby influencing therapeutic responses.

Interestingly, in the validation cohort, patients with the *GALNT14*-rs62139523 “non-A/G” genotype were significantly associated with a higher proportion of males (65.6% vs. 43.4% in patients with the “A/G” genotype) and poorly differentiated histology (20.8% vs. 7.1% in patients with the “A/G” genotype) (Table 3). This could be because, while CRC occurs in both sexes, it exhibits sex differences [53]. Current understanding indicates that sex impacts CRC incidence, clinicopathological features like poor differentiation status, therapeutic outcomes, and tolerance of anticancer treatments [53]. Specifically, tumor biology in CRC differs between males and females due to sex hormones and sex chromosomes influencing immunity and regulating key proliferative pathways through estrogens [54,55]. Additionally, significant sex-associated differences exist in the pharmacokinetics of 5-FU-based chemotherapy [56]. Females have been found to have lower plasma clearance and dose, while plasma life and area under the plasma concentration–time curve are higher compared with males. These differences can impact 5-FU treatment outcomes and toxicity, which is reported to be more extensive in females due to lower clearance of 5-FU, leading to higher plasma levels [57,58,59].

In the 5-FU catabolism and clearance process, the rate-limiting enzyme is DPYD, which converts more than 80% of 5-FU to DHFU in the liver [20]. The protein product of the *GALNT14* gene may serve oncogenic roles and regulate anti-cancer drug sensitivity in multiple cancers, including hepatocellular carcinoma [50], possibly through modulating downstream effector glycosylation. DPYD has been reported to be a downstream gene of p53 [60], which is a substrate of *O*-glycosylation [61]. Accordingly, alterations in the genotypes of *GALNT14*-rs62139523 may affect GALNT14 expression, thereby disturbing subcellular glycosylation status and response to 5-FU-mediated cytotoxicity [62]. However, further investigations are needed to clarify the detailed underlying mechanisms.

An issue highlighted by the results of this study is the unclear mechanism through which *GALNT14*-rs62139523 genotypes influence 5-FU-based adjuvant chemotherapy in stage II and III CRC patients after curative surgery. Although this SNP resides within the *GALNT14* gene, it is positioned downstream of the 3′ untranslated region of all identified and reported mRNA isoforms of *GALNT14*, suggesting that it may not directly impact the expression of the *GALNT14* gene. However, it is situated within the intron of another putative gene, *ENSG00000285984*, which encodes a putative long non-coding RNA (lncRNA). Unfortunately, the function of this lncRNA remains unknown at present.

In this study, we also identified *DNMBP*-rs10786578 genotypes as another potential candidate for predicting the efficacy of post-resection 5-FU-based adjuvant chemotherapy in stage II/III CRC patients. Although there was a tendency towards a favorable outcome in patients with the *DNMBP*-rs10786578 “C/T” genotype, it did not reach statistical significance in the validation cohort for either OS or PFS (Figure 3A,B). This might be due to the insufficient stringent *p*-value threshold criteria potentially leading to a false positive in this study. However, it is also possible that *DNMBP*-rs10786578 genotypes are indeed a genetic biomarker for predicting the efficacy of post-resection 5-FU-based adjuvant chemotherapy in stage II/III CRC patients, and the lack of statistical significance could be attributed to the limitations of this study, particularly the small sample sizes.

This study possesses several limitations, such as its retrospective design, a restricted number of cases, and its focus on a Chinese population from a single medical center. The utilization of the Axiom Genome-Wide TWB 2.0 Array Plate may have limited the identification of other potential genetic variants, given that only 686,463 SNPs are covered by this array. Additionally, our eligibility criteria confined treatments to 5-FU-based chemotherapies (mFOLFOX6, FOLFIRI, and XELOX) for interpretative clarity, potentially limiting generalizability to other non-5-FU regimens. Treatment decisions were influenced not only by clinical criteria but also by nonclinical considerations, such as physician discretion and patient preferences. Despite these limitations, the practical value of our study results for routine clinical practice is evident. Furthermore, as 5-FU-based regimens are also frequently used as adjuvant chemotherapeutic agents for other cancer types, such as pancreatic cancer [63], gastric cancer [64], and breast cancers [65], our findings may have future applications for patients with these cancers who are receiving 5-FU-based adjuvant chemotherapy.

## 4. Materials and Methods

### 4.1. Patients and Samples

A total of 226 patients diagnosed with intermediate stages of CRC, who underwent surgical tumor resection between 2003 and 2017 and subsequently received 5-FU-based adjuvant chemotherapy, were retrospectively included in this study. The chemotherapy regimens included modified folinic acid, 5-FU, oxaliplatin (mFOLFOX6, with biweekly courses of oxaliplatin 85 mg/m^2^, leucovorin 400 mg/m^2^, and 5-FU 400 mg/m^2^ bolus, followed by a 46-h intravenous infusion of 5-FU 2400 mg/m^2^), folinic acid, fluorouracil, irinotecan (FOLFIRI, with irinotecan 180 mg/m² and 5-FU 400 mg/m² bolus, then 5-FU 1200 mg/m²/day continuous infusion for 2 days), and capecitabine plus oxaliplatin (XELOX, with intravenous infusion of oxaliplatin 130 mg/m² accompanied by capecitabine 1700–2000 mg/m² orally for two weeks, repeated every 3 weeks for a total of 8 cycles). Among these patients, 31 were part of the exploration cohort, while the remaining 195 were included in the validation cohort. All patients were treated at the same medical center, ensuring uniformity in treatment protocols and follow-up procedures. Importantly, the patients in the exploration and validation cohorts were independent, with no overlap between the two groups. Tissue samples were retrospectively retrieved from the Research Specimen Processing Laboratory and Biobank at Linkou Chang Gung Memorial Hospital, approved by the Institutional Review Board (IRB) at Chang Gung Memorial Hospital (Approval numbers: 202001598B0 and 202102161B0), which waived the requirement for informed consent. Detailed clinicopathological data, including age, sex, carcinoembryonic antigen (CEA) levels, tumor location, size, margin status, depth of invasion, regional lymph node involvement, tumor differentiation, histology type, chemotherapy regimen, dates of CRC diagnosis, CRC-related death, last follow-up, and recurrence or metastasis, were meticulously collected. Overall survival (OS) was calculated from the date of surgery to the date of death or the last follow-up, while progression-free survival (PFS) was determined from the date of surgery to disease progression, as evidenced by imaging studies, or death from any cause. Staging followed the tumor–node–metastasis (TNM) classification per the American Joint Committee on Cancer (AJCC) 8th edition. CRCs with regional lymph node involvement (N1, 1 to 3 nodes; N2, ≥4 nodes) and tumor invasion (T1 to T4) without distant metastasis (M0) were classified as stage III. CRCs with at least muscularis propria invasion (≥T3) without lymph node involvement or distant metastasis were classified as stage II. The intermediate stage encompassed both stage II and stage III CRC patients.

### 4.2. Genotyping Using TWB 2.0 Chip

In the exploratory cohort, genomic DNA from patients was meticulously extracted from patient-derived tissues using the QIAamp DNA Mini Kit (QIAGEN, Hilden, Germany, Cat: 51306), strictly adhering to the manufacturer’s instructions. To guarantee the quality and suitability of the isolated genomic DNA for subsequent analysis, its integrity was assessed through agarose gel electrophoresis, and its quantity was precisely measured using spectrophotometry. Subsequently, 1 μg of genomic DNA underwent genotyping using the Axiom Genome-Wide TWB 2.0 Array Plate, processed at the National Center for Genomics Medicine, Academia Sinica, Taipei, Taiwan. This array, a custom-designed Axiom GWAS array, features 686,463 SNPs specifically tailored to the genetic characteristics of the Han Chinese Taiwanese population, facilitating the construction of a population-specific genetic reference for this demographic [25]. Sample hybridization to the microarrays and the subsequent washing procedure strictly adhered to the manufacturer’s protocol, maintaining all parameters at their default settings throughout the analysis to ensure consistency and reliability of the results. The genetic variations were referenced to the GRCh38 reference genome.

### 4.3. Genotyping Using Polymerase Chain Reaction (PCR) Followed by Direct Sequencing

Genomic DNA from patients in the validation cohort was meticulously extracted as described above and subjected to PCR-based amplification of specific DNA regions containing the SNP candidates identified in the GWAS, followed by direct sequencing for genotyping. Primers *DNMBP*-rs10786578_F: 5′-TAGTTGCCCAACAATCCTCA-3′, *DNMBP*-rs10786578_R: 5′-AGCCCTGGGACGAAGGATAA-3′, *GALNT14*-rs62139523_F: 5′-TGTCTCCCTTGTAGGGTTGT-3′, and *GALNT14*-rs62139523_R: 5′-TCAGACTGGCATCTCAGGGA-3′ were used in this study. Subsequently, direct Sanger’s sequencing was employed to examine the genotype of each SNP.

### 4.4. Statistical Analysis

The characteristic data were expressed as percentages (%) for categorical variables, means ± standard deviation for continuous variables with a normal distribution, and median (range) for continuous variables with a non-normal distribution, as determined via the Kolmogorov–Smirnov method. Group comparisons utilized the Chi-square or Fisher’s exact tests for categorical data, while the two-sample Student’s *t*-test or Mann–Whitney U test was employed for continuous variables with or without normal distribution. Kaplan–Meier survival analysis, along with the log-rank test, was employed for outcome comparisons. Clinicopathological parameters underwent univariate and multivariate analysis using the Cox proportional hazards regression model to identify predictive factors for OS and PFS. Sensitivity analysis of the *GALNT14*-rs62139523 genotyping test was conducted in different subgroups using the Cox model. Instances where patients were lost to follow-up were treated as censored data. A significance level of *p* < 0.05 denoted statistical significance. All statistical analyses were carried out using SPSS version 26.0 software (SPSS Inc., Chicago, IL, USA).

## 5. Conclusions

In conclusion, this study conducted a comprehensive GWAS analysis to explore genetic predictors of 5-FU-based adjuvant therapy in intermediate-stage CRC patients, identifying *GALNT14*-rs62139523 and *DNMBP*-rs10786578 genotypes as potential predictors. Subsequent validation in a larger cohort of 195 patients underscored the predictive significance of *GALNT14*-rs62139523 genotypes, particularly the “A/G” genotype, in terms of OS and PFS. Notably, this predictive association was robust across various subgroups, except for specific demographic and clinical parameters such as age < 58 years old, CEA ≤ 2.5 ng/mL, tumor diameter > 44.0 mm, and tumor free margin ≥ 50 mm. These findings emphasize the promising role of *GALNT14*-rs62139523 “A/G” genotype as a biomarker for predicting favorable outcomes in intermediate-stage CRC patients undergoing surgical resection followed by 5-FU-based adjuvant chemotherapy, with attention to specific subgroup characteristics. Our findings have the potential to inform personalized treatment plans through predicting patient responses to 5-FU-based chemotherapy, thereby improving efficacy and reducing side effects. Additionally, genetic profiling facilitates risk stratification, allowing clinicians to identify high-risk patients for aggressive treatment and closer monitoring, ultimately enhancing patient management and outcomes. While our findings hold promise in predicting outcomes for stage II/III CRC patients receiving curative resection followed by adjuvant 5-FU-based chemotherapy, further research is warranted. This should include longitudinal studies to validate these findings across diverse populations and experimental studies to elucidate the functional mechanisms underpinning this association. Our findings, in concert with ongoing research, underscore the importance of personalized medicine in the evolving landscape of CRC treatment and prognosis.

## Figures and Tables

**Table 1:** Basic clinicopathological factors of patients included in the exploration cohort.

Variable	Good Prognosis (*n* = 17)	Poor Prognosis (*n* = 14)	*p*
Gender, male, n (%)	6 (35.3%)	7 (50.0%)	0.646
Age, year, mean ± SD	63.7 ± 10.5	66.9 ± 10.5	0.320
CEA, ng/mL, median (range)	1.8 (0.5–6.5)	34.6 (0.5–1332.0)	**0.002**
Tumor diameter, mm, mean ± SD	40.1 ± 12.8	50.3 ± 12.1	0.052
Tumor free margin, mm, mean ± SD	62.9 ± 42.3	33.6 ± 21.6	0.072
Tumor invasion, pT4, n (%)	3 (17.7%)	6 (42.9%)	0.254
Lymph node metastasis, yes, n (%)	16 (81.7%)	12 (64.8%)	0.859
Tumor differentiation, poor, n (%)	1 (5.9%)	2 (14.3%)	0.859
Tumor location, left-sided, n (%)	8 (47.1%)	11 (78.6%)	0.821
Overall survival, month, median (range)	67.0 (61.0–80.0)	18.0 (4.0–22.0)	**<0.001**
Progression-free survival, month, median (range)	67.0 (61.0–80.0)	8.5 (1.0–14.0)	**<0.001**

Bold values indicate statistical significance *p* < 0.05. CEA, carcinoembryonic antigen; SD, standard deviation.

**Table 2:** Candidate SNP genotype correlated with therapeutic efficacy of 5-FU-based regimen in CRC patients.

Chr	Start	dbSNP_ID	Gene	Functional Consequence	Ref	Alt	Good Prognosis (*n* = 17)			Poor Prognosis (*n* = 14)			*p*-Value
							Ref	Het	Alt	Ref	Het	Alt	
2	30890589	rs62139523	*GALNT14*, *ENSG00000285984*	Non-coding transcript variant	G	A	2	14	1	2	1	11	**0.0000585**
2	43643229	rs13408620	*PLEKHH2*	Intron variant	G	A	1	0	16	0	10	4	0.0001186
2	43638639	rs6544681	*PLEKHH2*	Intron variant	T	G	16	0	1	4	10	0	0.0001186
2	43637387	rs6544679	*PLEKHH2*	Intron variant	A	G	16	0	1	4	10	0	0.0001186
3	59825736	rs689497	*FHIT*	Intron variant	G	T	0	15	2	2	2	10	0.0001893
3	1665346	rs13078044	-	Intergenic	T	C	4	13	0	10	1	3	0.0003870
3	61797496	rs657813	*PTPRG*	Intron variant	G	A	4	13	0	8	1	5	0.0002635
5	26261937	rs28794939	-	Intergenic	G	A	0	0	17	1	9	4	0.0001281
5	4611800	rs13171304	-	Intergenic	A	G	0	10	7	8	6	0	0.0003601
6	149149120	rs62425913	*LOC124901426*	Intron variant	A	G	17	0	0	4	8	2	0.0001281
7	129784611	rs10259447	-	Intergenic	T	C	10	2	5	1	12	1	0.0001992
8	135197628	rs984449	-	Intergenic	G	A	0	11	6	8	1	5	0.0002907
10	99930293	rs10786578	*DMNBP*, *DNMBP-AS1*	Synonymous variant	C	T	0	15	2	6	1	7	**0.0000285**
11	131650003	rs10791160	*NTM*	Intron variant	C	A	17	0	0	5	8	1	0.0004530
12	61646586	rs7133799	-	Intergenic	A	C	1	7	9	10	3	1	0.0004965
13	67569545	rs7318264	-	Intergenic	C	T	12	2	3	1	11	2	0.0004109
16	11584433	rs2868424	*LITAF*	Intron variant	T	A	0	16	1	6	3	5	0.0001637
16	54175924	rs2542671	-	Intergenic	T	C	12	2	3	1	11	2	0.0004109
17	45990481	rs2435213	*MAPT*	Intron variant	G	T	15	1	1	3	10	1	0.0004965
19	44823511	rs1871045	-	Intergenic	C	T	7	10	0	1	4	9	0.0003474

Bold values indicate the SNPs with the highest statistical significance. Abbreviations: Chr, chromosome; Start, the start site of the variant nucleotide (GRCh38); Ref, reference sequence; Alt, alteration sequence; Het, heterozygote.

**Table 3:** Basic clinicopathological factors of patients included in the validation cohort.

Variable	All Patients (*n* = 195)	*DNMBP*-rs10786578			*GALNT14*-rs62139523		
		“C/T” (*n* = 109)	“non-C/T” (*n* = 86)	*p*	“A/G” (*n* = 99)	“non-A/G” (*n* = 96)	*p*
Gender, male, n (%)	106 (54.4%)	62 (56.9%)	44 (51.2%)	0.426	43 (43.4%)	63 (65.6%)	**0.002**
Age, year, mean ± SD	57.2 ± 11.6	58.1 ± 10.8	56.0 ± 12.4	0.221	57.0 ± 11.0	57.3 ± 12.2	0.851
CEA, ng/mL, median (range)	2.5 (0.5–1332.0)	2.5 (0.5–107.4)	2.6 (0.5–1332.0)	0.391	2.2 (0.5–505.5)	2.8 (0.5–1332.0)	0.158
Tumor diameter, mm, mean ± SD	46.6 ± 20.9	44.8 ± 20.5	48.8 ± 21.3	0.186	46.0 ± 21.7	47.2 ± 20.1	0.677
Tumor free margin, mm, mean ± SD	60.9 ± 49.0	61.6 ± 47.8	60.0 ± 50.1	0.836	66.8 ± 50.7	54.8 ± 46.8	0.088
Tumor invasion, pT4, n (%)	45 (23.1%)	26 (23.9%)	19 (22.1%)	0.772	21 (21.2%)	24 (25.0%)	0.530
Lymph node metastasis, yes, n (%)	184 (94.4%)	102 (93.6%)	82 (95.3%)	0.595	96 (97.0%)	88 (91.7%)	0.109
Tumor differentiation, poor, n (%)	27 (13.8%)	13 (11.9%)	14 (16.3%)	0.382	7 (7.1%)	20 (20.8%)	**0.005**
Tumor location, left-sided, n (%)	148 (75.9%)	80 (73.4%)	68 (79.1%)	0.358	72 (72.7%)	76 (79.2%)	0.293

Bold values indicate statistical significance *p* < 0.05. CEA, carcinoembryonic antigen; SD, standard deviation.

**Table 4:** Cox proportional hazard analysis of clinicopathological and genotypic parameters for OS and PFS in 195 CRC patients included in the validation cohort.

Variable	Univariate Analysis			Multivariate Analysis		
	HR	95% CI	*p*	HR	95% CI	*p*
**For OS**						
*DNMBP*-rs10786578, “C/T” = 1	0.617	0.339–1.123	0.114			
*GALNT14*-rs62139523, “A/G” = 1	0.326	0.167–0.638	**0.001**	0.397	0.200–0.787	**0.008**
Age, per year increase	1.016	0.989–1.044	0.242			
Gender, male = 1	1.333	0.723–2.456	0.357			
Tumor diameter, per mm increase	0.994	0.979–1.009	0.451			
Tumor free margin, per mm increase	0.993	0.985–1.001	0.082			
Tumor invasion, pT4 = 1	2.252	1.213–4.183	**0.010**	2.180	1.128–4.213	**0.022**
Lymph node metastasis, yes = 1	0.326	0.137–0.776	**0.011**	0.238	0.097–0.587	**0.002**
Tumor differentiation, poor = 1	1.235	0.550–2.776	0.609			
CEA, per ng/mL increase	1.005	1.003–1.008	**<0.001**	1.004	1.002–1.006	**<0.001**
Tumor location, left-sided = 1	0.762	0.391–1.484	0.424			
**For PFS**						
*DNMBP*-rs10786578, “C/T” = 1	0.716	0.424–1.209	0.211			
*GALNT14*-rs62139523, “A/G” = 1	0.434	0.248–0.759	**0.003**	0.451	0.255–0.795	**0.006**
Age, per year increase	1.007	0.984–1.030	0.580			
Gender, male = 1	1.354	0.792–2.315	0.268			
Tumor diameter, per mm increase	0.985	0.971–0.999	**0.040**	0.975	0.959–0.992	**0.003**
Tumor free margin, per mm increase	0.994	0.988–1.001	0.099			
Tumor invasion, pT4 = 1	1.813	1.034–3.179	**0.038**	2.398	1.323–4.346	**0.004**
Lymph node metastasis, yes = 1	0.565	0.225–1.419	0.224			
Tumor differentiation, poor = 1	1.305	0.639–2.664	0.465			
CEA, per ng/mL increase	1.003	1.002–1.004	**<0.001**	1.003	1.002–1.005	**<0.001**
Tumor location, left-sided = 1	0.728	0.407–1.300	0.283			

Bold values indicate statistical significance *p* < 0.05. OS, overall survival; PFS, progression-free survival; CEA, carcinoembryonic antigen; HR, hazard ratio; CI, confidence interval.

## Data Availability

The authors declare that all relevant data of this study are available within the article or from the corresponding author on reasonable request.
